# Basil as a Green Alternative to Synthetic Additives in Clean Label Gilthead Sea Bream Patties

**DOI:** 10.3390/foods15020198

**Published:** 2026-01-06

**Authors:** Branislav Šojić, Sandra Zavadlav, Danijela Bursać Kovačević, Nadežda Seratlić, Sanja Vojvodić, Predrag Ikonić, Tatjana Peulić, Nemanja Teslić, Miloš Županjac, Branimir Pavlić

**Affiliations:** 1 Faculty of Technology Novi Sad, University of Novi Sad, Bulevar Cara Lazara 1, 21000 Novi Sad, Serbiaseratlicnadezda@gmail.com (N.S.);; 2 The Department of Food Technology, Karlovac University of Applied Sciences, Trg J. J. Strossmayera 9, 47000 Karlovac, Croatia; 3 Faculty of Food Technology and Biotechnology, University of Zagreb, Pierottijeva 6, 10000 Zagreb, Croatia; 4 Institute of Food Technology, University of Novi Sad, Bulevar Cara Lazara 1, 21000 Novi Sad, Serbia

**Keywords:** gilthead sea bream, *Ocimum basilicum* L., essential oil, antioxidant activity, oxidative stability, spoilage indicators

## Abstract

This study investigated the effectiveness of basil (*Ocimum basilicum* L.) extract obtained by hydrodistillation (EO) and lipid extract (LE) obtained via supercritical fluid extraction in preserving the quality of ground fish patties during refrigerated storage. Gilthead sea bream (*Sparus aurata*) patties were formulated with varying concentrations of EO and LE and evaluated over three days at 4 °C. The chemical composition of the extracts, analyzed by GC-MS, revealed linalool, eucalyptol, and τ-cadinol as dominant bioactive compounds, with EO richer in monoterpenes and LE in sesquiterpenes. Both extracts significantly reduced lipid oxidation (TBARS) and protein oxidation (thiol content), with the strongest antioxidative effect observed in patties containing 0.150 µL/g of LE. Color parameters (L*, a*, b*, Δ*E*) were moderately influenced, without adverse effects on product appearance. pH and water activity values remained stable across treatments, while total volatile basic nitrogen (TVB-N) levels confirmed delayed spoilage in extract-treated patties. Results highlight the potential of basil extracts, especially LE obtained by SFE, as effective natural antioxidants in fish-based products. These findings support the development of clean-label, health-promoting products tailored to individual needs, and show that ground fish porridge has promise as a viable material for the production of innovative seafood products.

## 1. Introduction

In recent years, consumers have increasingly favored “clean label” food products—those with natural, simple ingredients, free from synthetic additives, and minimally processed. This tendency indicates an increasing consumer interest in foods that, beyond supplying basic nutritional value, also offer added health-promoting effects derived from bioactive compounds with antioxidant, antimicrobial, and anti-inflammatory activities [1]. As a result, incorporating plant-based extracts rich in bioactives into food formulations has attracted attention [2]. This movement supports sustainable food systems and personalized nutrition, emphasizing transparency, natural origins, and functional benefits. There is particular interest in animal-based products enhanced with natural additives that preserve sensory and technological qualities while improving health-promoting potential. These innovations meet consumer expectations for minimally processed yet functionally enriched foods that support wellness and preventive health [3,4]. Fish stands out in the clean label and functional food movement. Fish, being rich in high-quality protein, omega-3 fatty acids, and essential micronutrients, is widely recognized as a beneficial component of health-supporting diets [5]. However, its high perishability—due to water activity, enzymatic action, and susceptibility to oxidation and microbial spoilage—poses challenges for storage, distribution, and consumer acceptance. In response to this, researchers are increasingly exploring ways to transform fresh fish into value-added or intermediate products. Such material may serve as a promising candidate for future evaluation as a 3D food printing material. This enables improved portioning, nutritional customization, and extended shelf life while maintaining sensory and functional properties. It is especially useful for incorporating bioactive ingredients and tailoring products to specific dietary or health needs [6].

Today, a diverse range of plants is being studied as natural additives for functional foods, aiming to improve their nutritional content as well as extend shelf life. Among these plants, basil (*Ocimum basilicum* L.) has gained significant attention owing to its abundant bioactive constituents and proven antioxidant and antimicrobial properties [7]. Basil essential oil, characterized by key components such as linalool and eugenol, has been successfully incorporated into fish-based products in several studies [8]. For example, the addition of basil essential oil, combined with rosemary essential oil, to Atlantic mackerel fillets has significantly reduced microbial growth and lipid oxidation during refrigerated storage, thereby enhancing both safety and sensory quality [9]. Despite its potential, the incorporation of basil essential oil into fish patties has been little explored, highlighting a gap in the literature and the need for additional investigation, particularly given the rising preference for natural, clean-label products [10].

Based on the considerations above, the present work was designed to assess the use of basil (*Ocimum basilicum* L.) essential oil as a natural additive in fish products, with special attention to its bioactive, antioxidant properties and its impact on oxidation- and spoilage-related quality indicators. In particular, the study aimed to evaluate and compare basil essential oil (EO) obtained through hydrodistillation and lipid extract (LE) derived from supercritical fluid extraction (SFE), in terms of their chemical composition and antioxidant performance in ground fish patties under refrigerated storage. Preservation efficacy was examined via physicochemical parameters (pH, water activity, and color), markers of lipid and protein oxidation (TBARS and thiol content), and spoilage indicators (TVB-N). Ultimately, the research sought to support the development of natural, clean-label additives to enhance the shelf life, safety, and visual quality of fish-based products.

## 2. Materials and Methods

### 2.1. Preparation of Fish Fillet

For the main experiment, 38 samples of gilthead sea bream (*Sparus aurata*, *Linnaeus*, 1758) were caught at the Veli Bok fish farm during the same night along the Croatian Adriatic coast, between the town of Cres on the island of Cres and the Rabac coast. Farm Veli Bok is located in the bay of Veli Bok on the northwest side of the island of Cres at the geographical position φ = 45°01.7′ N, λ = 014°21.1′ E. All cages are 22 m in diameter, and the farm is situated where the sea depth is 50–60 m. Fish farming is based on a low stocking density of 120,000–130,000 fish per cage.

The fish were fed exclusively with diets free of hemoglobin, antibiotics, and genetically modified organisms (GMOs). Fresh gilthead sea bream, averaging 550 ± 25 g in weight, were kept on ice and transported in Styrofoam containers filled with crushed ice to the processing facility (Orada Adriatic d.o.o., Rijeka, Croatia). At the facility, post-rigor fish were decapitated, eviscerated, and filleted prior to analysis and freezing. Fresh fillets were cleaned to be boneless and represented the highest segment of gilthead sea bream fillets. Fillets with skin were individually quick frozen (IQF) in a stream of −45 °C cold air, with characteristic freezing times of 15 min (OctoFrost IQF Freezer, OctoFrost Group, Malmö, Sweden). Frozen samples were transported to the laboratory in Styrofoam boxes filled with crushed ice. Upon arrival at the laboratory (Institute of Food Technology, Novi Sad, Serbia), a total of 76 frozen gilthead sea bream fillets (average weight 175 ± 5 g), without skinning, were stored for 12 h in a refrigerator at 4 ± 1 °C until processing and analysis, which took place within 3 days. The average filet length of the filets was 26 ± 0.55 cm, and the thickness was 2.50 ± 0.05 cm.

### 2.2. Plant Material

Basil plants were grown in agricultural fields in Bačko Novo Selo and hand-harvested at peak maturity. The processing and handling procedures are detailed in a previous study [11]. After collection, the plant material was kept in paper bags at ambient temperature until required for analysis.

### 2.3. Chemicals

2-Thiobarbituric acid, Trichloroacetic acid; Butylhydroxytoluene (BHT), n-Hexane, 1,1,3,3-Tetraethoxypropane, Ellmans reagent (DTNB), and Bovine serum albumin (BSA) all of analytical grade were purchased from Sigma-Aldrich GmbH (Taufkirchen, Germany). L-cysteine of analytical grade was purchased from Fisher Scientific (Loughborough, UK). All other chemicals used were of analytical reagent grade. Commercial carbon dioxide (Messer, Novi Sad, Serbia), purity > 99.98%, was used for laboratory supercritical fluid extraction. All other chemicals were of analytical reagent grade.

### 2.4. Hydrodistillation (HD) and Supercritical Fluid Extraction (SFE) of Basil

A Clevenger-type apparatus was used for hydrodistillation of basil essential oil (EO) according to the official procedure of Ph. Eur. IX [12]. Twenty grams of basil were mixed with 400 mL of distilled water and distilled for 2 h. Basil EO isolation was performed in three repetitions, and the yield was expressed as % (*v*/*w*). Collected basil samples were stored at 4 °C to prolong the stability of the isolated EOs.

Furthermore, environmentally oriented supercritical fluid extraction (SFE) was performed to obtain the basil liquid extract (LE). The equipment used for SFE of basil was HPEP, NOVA-Swiss, Effretikon, Switzerland, following the procedure of Zeković et al. [13]. The selected extraction parameters for basil LE were: pressure of 300 bar, CO_2_ flow rate of 0.2 kg CO_2_/h, temperature of 60 °C, and extraction time of 4 h. Additionally, 50 g of basil was used to obtain LE, which was stored at 4 °C in glass vials until further analysis.

### 2.5. Characterization of Basil Extracts Obtained by HD and SFE

The chemical profile of basil essential (EO) and lipid extract (LE) was determined by GC-MS analysis using GC system (7890A, Agilent Technologies, Santa Clara, CA, USA) coupled with an MS detector (5975C, Agilent Technologies, USA) [14]. Basil EO and LE were mixed with methylene chloride. Then, 1 µL of each sample was injected into a capillary column (30 m × 0.25 mm, 0.25 µm, 19091S-433UI HP-5MSUI, Agilent Technologies, USA) in spitless mode. Helium (>99.9997%) was used as the carrier gas at a flow rate of a mobile phase with 2 mL/min of flow rate. The initial temperature was set at 60 °C for 30 min, then increased at 3 °C/min to 150 °C. Subsequently, the temperature was raised to 250 °C at a rate of 20 °C/min and held for 5 min. The final 5 min were maintained at 250 °C, which was also the injector temperature. Identification was performed using the NIST database (version 2005) of MS spectra and literature databases [15]. Results were presented as relative content (%), and the dominant compounds (eucalyptol, germacrene D, and geraniol) were quantified (mg/g) using analytical standards.

### 2.6. Preparation of “Clean Label” Ground Fish Patties

**Figure 1:**
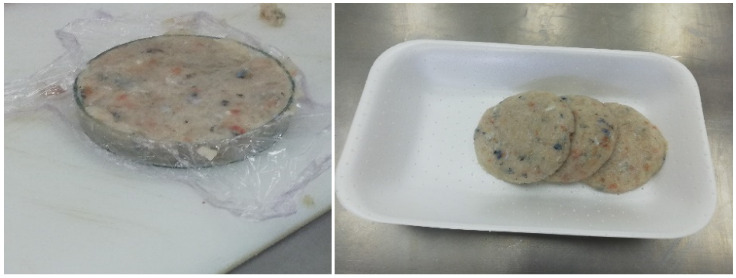
Prepared “clean-label” fish patties with a weight of 60 g, a diameter of 9.0 cm, and a thickness of 1.5 cm.

Fillets were double-ground using an 8 mm plate grinder and kept at 4 °C until further processing. Clean-label patties were prepared using a minimal-ingredient approach, avoiding synthetic additives and prioritizing natural components. The basic formulation included 98% fish meat and 2% salt. Five treatments were applied with basil essential oil (EO) or lipid extract (LE): GFP1 (0.075 µL/g EO), GFP2 (0.150 µL/g EO), GFP3 (0.075 µL/g LE), GFP4 (0.150 µL/g LE), and GFPC as the control. For each treatment, three patties were produced. Patties (60 g; 9.0 cm diameter; 1.5 cm thick) were manually molded for 2 min (Figure 1).

The temperature was maintained below 5 °C throughout the entire patty manufacturing process. Patties were placed in polypropylene trays, overwrapped with an oxygen-permeable polyvinyl chloride film, and stored at 4 °C for 3 days. Samples were taken at 0, 1st, 2nd, and 3rd day of refrigerated storage, with three randomly selected ground fish patties from each treatment.

The storage period of 3 days at 4 °C was selected to simulate the typical shelf life of fresh, minimally processed fish patties stored under aerobic conditions and without the use of synthetic preservatives. Such products are highly susceptible to rapid oxidative and spoilage-related changes, particularly during the early stages of refrigerated storage. Therefore, this time frame is appropriate for evaluating the initial effectiveness of natural antioxidants under conditions relevant to fresh retail products and pilot-scale formulation studies.

### 2.7. Physicochemical Analysis

The pH was measured in three samples from each group of patties using a digital pH meter (TESTO 205, Testo SE & Co, Baden-Württemberg, Germany) with automatic temperature compensation and a calibrated electrode, which was inserted directly into fish patty for reading.

Water activity (aw) was determined using a LabSwift Portable Water Activity Meter (Novasina AG, Lachen, Switzerland) with direct readings. Measurements were performed in triplicate, and the average value was obtained after the samples were uniformly packed in a measuring chamber.

Surface color measurements of the fish patties were performed using a MINOLTA Chroma Meter CR-400 (Minolta Co., Ltd., Osaka, Japan). The color parameters evaluated included lightness (L*), redness (a*), and yellowness (b*). Measurements were taken with an 8 mm diameter aperture and a 2° standard observer under illuminant D-65.

Total color differences (Δ*E*) between treatments GFP1, GFP2, GFP3, and GFP4 (*T*) and the control GFPC (*C*) were calculated as:

$$∆E=\sqrt[{2}]{{{(L}_{T}^{*}-L_{C}^{*})}^{2}+{{(a}_{T}^{*}-a_{C}^{*})}^{2}+{{(b}_{T}^{*}-b_{C}^{*})}^{2}}$$

where $L_{T}^{*}\;$ and $L_{C}^{*}$ represent the lightness values of the treated (*T*) and control (*C*) samples, respectively; $a_{T}^{*}$ and $a_{C}^{*}$ represent the chromaticity values on the red-green axis, and $b_{T}^{*}$ and $b_{C}^{*}$ represent the chromaticity values on the yellow-blue axis for the treated and control samples, respectively. Color measurements were performed on three samples from each group of patties in duplicate.

### 2.8. Lipid Oxidation

As outlined previously, lipid peroxidation in the ground fish patties was quantified via the 2-thiobarbituric acid reactive substances (TBARS) method [16]. Results were expressed as mg malondialdehyde (MDA) per kg of patties. Three samples from each treatment were analyzed in duplicate.

### 2.9. Protein Oxidation

The extent of protein oxidation in ground fish patties was determined by measuring free thiol content according to a previously described method [17]. Results were expressed as nmol of thiol per mg of protein and three samples per group were analyzed in duplicate.

### 2.10. Total Volatile Basic Nitrogen (TVB-N)

Fish is a perishable product, and during storage, the actions of microorganisms and endogenous enzymes cause chemical compositional changes. Total volatile basic nitrogen (TVB-N) is used as a biomarker of protein and amine degradation. TVB-N was determined according to the procedure by Zavadlav et al. [18]. TVB-N was evaluated in three samples from each group of patties, in duplicate.

### 2.11. Statistical Analysis

Data analysis was performed using STATISTICA 13.0 (TIBCO Software Inc., Palo Alto, CA, USA). All results are presented as mean ± standard deviation (SD). One-way ANOVA was applied to assess differences among treatments, followed by Duncan’s multiple range test for pairwise comparisons at *p* ≤ 0.05.

## 3. Results and Discussion

### 3.1. Chemical Profiling Basil EO and LE

Given the high perishability of fresh fish patties and their short commercial shelf life under aerobic refrigeration, the present study focused on short-term storage to capture early oxidative and spoilage-related changes. This approach enables the assessment of antioxidant efficacy during the most critical period for quality deterioration in clean-label fish products. According to Table 1, slight differences in the chemical profile, expressed as relative percentages, between basil essential oil obtained by hydrodistillation (HD) and light extract (LE) recovered by supercritical fluid extraction (SFE) were determined by GC-MS. Among the common group of terpenoid compounds, oxygenated monoterpenes were the most dominant, followed by sesquiterpene hydrocarbons, oxygenated sesquiterpenes, and monoterpene hydrocarbons in both analyzed samples. Besides terpenes, another compound present in the basil essential oil sample was 1,5,5-trimethyl-6-methylene-cyclohexene (0.07%), while in the LE sample, a few more non-terpenoid compounds were identified (lavender lactone, 2,6-dimethyl-3,7-octadiene-2,6-diol, and 1,5,5-trimethyl-6-methylene-cyclohexene), with a total relative percentage of 1.46%. The main compound, linalool, was present in both samples, and the impact of the applied technique was evident in the linalool content: HD isolated 66.95%, while SFE extracted 60.80%. By investigating three different varieties of basil, it was confirmed that essential oil from *O. basilicum* var. *thyrsiflora* contains 68% linalool as the dominant terpenoid compound [19]. The same oxygenated monoterpene was found at half that content (31.6%) in the study by Stanojević et al. [16]. The results in this manuscript are in strong agreement with the study by Raina et al. [20], in which 64.13% linalool was measured in basil EO.

Along with traditional hydrodistillation, SFE was performed at 300 bar, 60 °C, and 0.2 kg CO_2_/h: these parameters were chosen based on the highest extraction yield reported by Zeković et al. [13]. Filip et al. [21] conducted a fractionation process using SFE to isolate and characterize terpenoid compounds from sweet basil. Different temperatures (40 and 50 °C) and pressures (100 to 300 bar) with a flow rate of 5.7 kg CO_2_/h were applied. The most dominant group of terpenoid compounds was oxygenated monoterpenes (22.94%), the same as in the LE sample in this study. The main compounds in their work (linalool, eugenol, α-bergamotene, germacrene D, γ-cadinene, δ-cadinene, β-selinene and spathulenol) showed that slight differences in chemical profile can be observed when compared with the chemical profile identified in this study, possibly due to different extraction conditions. Furthermore, a much higher amount of linalool in the LE sample (60.80%) was measured than in the study by Filip et al. [21] (20.71%). A similar pattern was also observed in the work of Arranz et al. [19], where linalool (27.81%) was the main compound in the SFE extract obtained under 30 MPa pressure, 40 °C, a CO_2_ flow rate of 60 g/min, and an extraction time of 5 h.

In the same group of terpenoid compounds, eucalyptol was isolated at 6.50% in the HD sample and 3.68% in the LE sample. In the next most dominant group, sesquiterpene hydrocarbons were extracted at 10.80% in the conventional EO sample, which had a total content of 99.23%. Within this group, τ-cadinol was notable at 5.19%. A slightly lower content of τ-cadinol (4.06%) in the LE sample made it the second most dominant compound in the extract, where sesquiterpene hydrocarbons accounted for 16.13% of the total 99.05%. The LE sample obtained by SFE was also characterized by a high content of germacrene D (3.55%). The group of monoterpene hydrocarbons was less prevalent, with the lowest percentages of α-thujene, camphene, α-terpinene, p-cymene, and trans-β-ocimene identified in the EO sample. A similar pattern was observed in the LE samples, with sabinene, p-cymene, phellandrene, and γ-terpinene present in low amounts.

Considering the techniques applied, some inconsistencies in content were observed. In the case of basil EO, the absence of lavender lactone, epoxylinalol, and carvone was noted, while in LE, α-thujene, camphene, α-terpinene, trans-β-ocimene, and menthol were missing compared to the EO. The composition of LE included a higher content of sesquiterpene hydrocarbons and a significantly lower content of monoterpene hydrocarbons compared to the chemical profile of the EO sample. In the study by Zeković et al. [22] the comparison of techniques regarding the higher content of linalool as the main compound (HD—50.09%, SFE—16.60%) was consistent with the linalool content in the HD (66.95%) and SFE (60.80%) samples, but with a more pronounced difference in results. Several authors have isolated and compared basil EO with basil LE from SFE in terms of chemical profile [13,23,24]. The same pattern of different chemical compositions of EO and LE obtained by SFE from sweet basil cultivated in Italy was observed in our research [23]. τ-Cadinol (27.5%) was the major compound in essential oil, which was significantly higher than the 5.19% in the EO sample and 4.06% in the LE sample from SFE obtained in our study. Conversely, the content of linalool did not match this work and was lower in both samples (HD—4% and LE—23.2%). Furthermore, the amounts of eucalyptol (10%) and germacrene D (5.6%) were higher in the LE sample compared to the amounts measured in our work, likely due to different conditions (temperature 50 °C, flow rate of 4.8 kg CO_2_/h, pressure of 15 MPa, and 95% ethanol as co-solvent).

Furthermore, for precise insight into the concentration of certain substances, quantitative analysis was performed using standard compounds (Table 2). Eucalyptol, the second most dominant oxygenated monoterpene in both samples, was found at 36.65 mg/g in the EO sample and 12.21 mg/g in the LE sample. In contrast, the content of geraniol was higher in LE (14.97 mg/g) than in EO (10.38 mg/g). Moreover, germacrene D was present at a higher concentration in the EO sample (15.99 mg/g) compared to the LE sample (6.3 mg/g). The quantitative content of these three compounds was also analyzed in EO and LE samples from SFE by Filip et al. [25]. According to the amounts of terpenoid compounds in EO (eucalyptol 25.0 g/kg, geraniol 15.3 g/kg, germacrene D 15.2 g/kg) and LE (eucalyptol 2.2 g/kg, geraniol 11.3 g/kg, germacrene D 53.6 g/kg), the content of eucalyptol was noticeably lower, geraniol was almost the same, and the content of germacrene D in essential oil was the same as in our EO sample, but in LE it was significantly higher. Elgndi et al. [26] analyzed the chemical profile of EOs and LE samples obtained by SFE from *S. montana*, *C. sativum*, and *O. basilicum*. SFE was performed at extraction parameters (300 bar and 40 °C, 0.2 kg CO_2_/h) similar to those we applied. The quantitative analysis showed that the content of eucalyptol (32.10 mg/g in HD and 12.10 mg/g in SFE) was consistent with our study, while the opposite was observed for geraniol (HD—22.00 mg/g and SFE—9.1 mg/g), where EO had a higher percentage of geraniol than LE.

The EO was richer in volatile oxygenated monoterpenes (e.g., linalool, eucalyptol), which are more prone to evaporation and partition preferentially into the headspace, contributing to early antioxidant and antimicrobial effects. In contrast, the LE obtained by SFE was enriched in less volatile, more lipophilic sesquiterpenes (e.g., germacrene D, τ-cadinol), which preferentially partition into the lipid phase of the fish matrix. This likely explains the stronger and more sustained inhibition of lipid oxidation observed for LE-treated patties, particularly at 0.150 µL/g. Because lipid oxidation products are known to promote secondary protein oxidation, the improved preservation of thiol groups in LE-treated samples is consistent with their enhanced lipid-phase protection. To further support this mechanistic link, correlation analysis between TBARS and thiol content has now been included in the revised manuscript, showing a significant negative correlation (*p* ≤ 0.05), thereby reinforcing the interdependence between lipid and protein oxidation in this system.

### 3.2. pH, Water Activity and Colorimetric Evaluation of Clean Label Ground Fish Patties During Storage

Measured pH values of the ground fish patties varied from 5.96 to 6.23 (Table 3). During the initial 24 h, a significant decline (*p* ≤ 0.05) was observed in the GFP2 and GFP4 samples, which may be attributed to the activity of lactic acid bacteria and subsequent accumulation of organic acids, particularly lactic acid. [27,28]. During the second day, a significant rise (*p* ≤ 0.05) in pH was observed for the GFPC, GFP1, and GFP3 treatments, which may be attributed to the buildup of alkaline compounds, including peptides, amines, and amino acids [29]. By the end of the storage period, the pH values for the GFPC, GFP1, GFP2, GFP3, and GFP4 treatments were measured to be 6.01, 5.96, 6.08, 6.05, and 6.00, respectively. Similar results have been reported in other studies [30,31]. The incorporation of basil EOs and LE did not have a significant (*p* ≥ 0.05) effect on the pH values at the end of the storage period, as there were no significant differences among the sample groups. This finding is consistent with results from a study on salmon and seaweed burgers, where the use of thyme and oregano EOs did not affect pH values [30]. It should be noted that pH values between 6.8 and 7.0 are generally considered acceptable for fish, while values above 7.0 indicate spoilage [32]. The initial a_w_ values of the ground fish patties ranged from 0.958 to 0.968 (Table 3). By the end of the storage period, significant changes (*p* ≤ 0.05) in the a_w_ values were observed, with values of 0.965, 0.969, 0.969, 0.964, and 0.966 for the GFPC, GFP1, GFP2, GFP3, and GFP4 treatments, respectively. The a_w_ values were similar for all samples at the end of the storage period. The use of basil EO and LE in the ground fish patties did not affect the a_w_ values.

The color parameters of ground fish patties, including L* (lightness), a* (redness), b* (yellowness), and Δ*E* (total color difference), were measured in this study. The recorded values ranged from 68.62 to 73.55 for L*, 0.06 to 2.31 for a*, 6.95 to 15.61 for b*, and 1.57 to 9.26 for Δ*E*. The addition of basil EO and LE to the ground fish patties did not have a significant influence on the L* and a* values (*p ≥ 0.05*) between treatments, indicating that the treatments did not negatively affect the perceived lightness or redness of the products.

Initially, higher values of a* (redness) were observed on day 0, but these values decreased by day 2. The loss of redness in fish products is likely due to oxidative reactions [33,34]. The b* (yellowness) value of the ground fish patties increased with storage time, indicating a shift toward a more yellowish color during storage, which is consistent with findings from other studies [35,36]. Total color difference (ΔE) was assessed according to the National Bureau of Standards (NBS) scale: 0–0.5 (trace), 0.5–1.5 (slight), 1.5–3.0 (noticeable), 3.0–6.0 (appreciable), and >12.0 (very much) [36]. In this study, the differences between treatments GFP1, GFP2, GFP3, and GFP4 compared to the control (GFPC) were appreciable throughout the storage period (Δ*E* > 3), suggesting perceptible color alterations, according to this standard.

This result indicates the effect of natural antioxidants and polyphenolic compounds present in basil EOs and LE, which have been reported to influence the color stability of fish and meat products [8,37,38]. While pH, water activity, and color parameters are commonly reported for fish products, the novelty of this study lies in demonstrating that basil essential oil (EO) and, notably, basil lipid extract (LE) obtained by supercritical fluid extraction can be incorporated into clean-label gilthead sea bream patties without adversely affecting these key physicochemical and visual quality attributes during refrigerated storage. To the best of our knowledge, such comparative data for EO versus SFE-derived LE in ground fish patties have not been previously reported. Furthermore, the results show that despite their distinct chemical profiles, both extracts maintained matrix stability while enabling effective oxidative protection, which is critical for the practical application of natural antioxidants in fish-based formulations. These findings provide new insight into the technological feasibility of using basil extracts—particularly SFE-derived LE—as functional clean-label additives that preserve quality without compromising appearance or basic physicochemical properties, thereby supporting their potential industrial applicability.

### 3.3. Lipid Oxidation of Clean Label Ground Fish Patties

**Figure 2:**
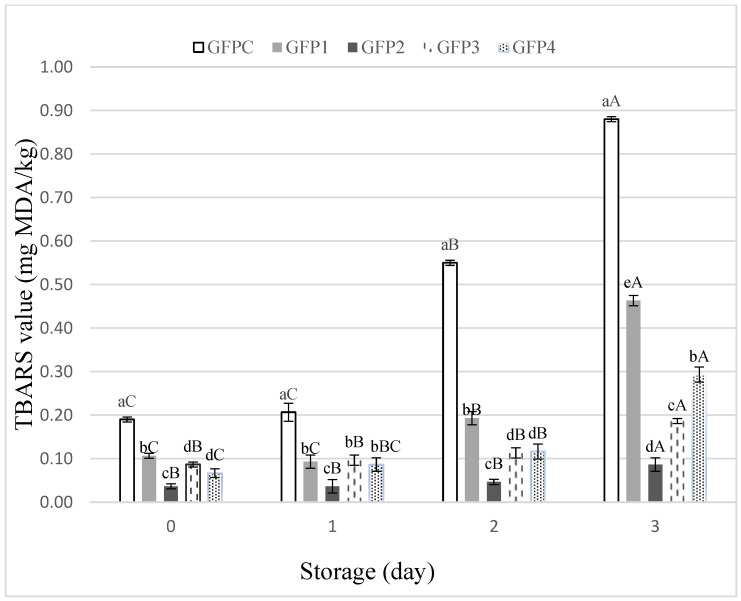
TBARS values of ground fish patties during storage (Different lower cases in superscripts (a–e) indicate difference (*p* ≤ 0.05) between treatments; Different Upper cases superscripts (A–C) indicate difference (*p* ≤ 0.05) between days of storage).

Lipid oxidation is a critical concern in fish products due to their high content of polyunsaturated fatty acids (PUFAs), which are highly susceptible to oxidative degradation [39,40]. The progression of lipid oxidation in clean label ground fish patties (GFP) was assessed using TBARS values, which measure malondialdehyde (MDA) concentration, a key indicator of lipid peroxidation. Figure 2 shows TBARS values for the control (GFPC) and different treatments (GFP1, GFP2, GFP3, GFP4) over three days of storage. As expected, TBARS values increased over time in all samples, reflecting ongoing lipid oxidation during storage. The control patties (GFPC) exhibited the highest TBARS value (0.15 mg MDA/kg), significantly greater than those of the treated samples (*p* ≤ 0.05), indicating that untreated patties were more prone to lipid oxidation. Among the treated samples, GFP1 and GFP4 showed moderate increases in TBARS values, while GFP2 and GFP3 maintained relatively low levels of lipid oxidation, suggesting that both basil EO and LE were effective in delaying oxidative processes during storage. The observed outcomes corroborate earlier research demonstrating the effectiveness of basil EO in enhancing oxidative stability in fish products [9]. Notably, TBARS levels remained below 0.5 mg MDA/kg for all treatments except the control over the storage period, aligning with the accepted sensory limit for rancid flavor detection [41]. These findings support the use of basil extracts as natural additives, with their antioxidant potential linked to the composition of EO and LE determined via GC-MS.

The most abundant compounds in the extracted oils were linalool (66.95% in HD, 60.80% in SFE), τ-cadinol (5.19% in HD, 4.06% in SFE), eucalyptol (6.50% in HD, 3.68% in SFE), and germacrene D (1.71% in HD, 3.55% in SFE). Linalool and τ-cadinol have been well-documented for their strong antioxidant properties, acting as free radical scavengers and inhibiting lipid peroxidation [39]. Similarly, eucalyptol has been shown to interrupt lipid oxidation chains by stabilizing lipid radicals, while germacrene D has demonstrated antimicrobial and antioxidant properties in other food preservation studies [39]. In the GFP2 treatment, the higher EO concentration (0.150 µL/g) likely provided a sufficient number of antioxidant molecules to effectively neutralize free radicals, preventing chain reactions of lipid peroxidation. Although the other treatments also showed satisfactory reductions in lipid oxidation, GFP2 stood out due to its high potency. This suggests that a lower amount of basil extract would be needed in product formulations, contributing to minimal increases in production costs and making it a more practical and cost-effective solution for industrial applications.

### 3.4. Protein Oxidation of Clean Label Ground Fish Patties

**Figure 3:**
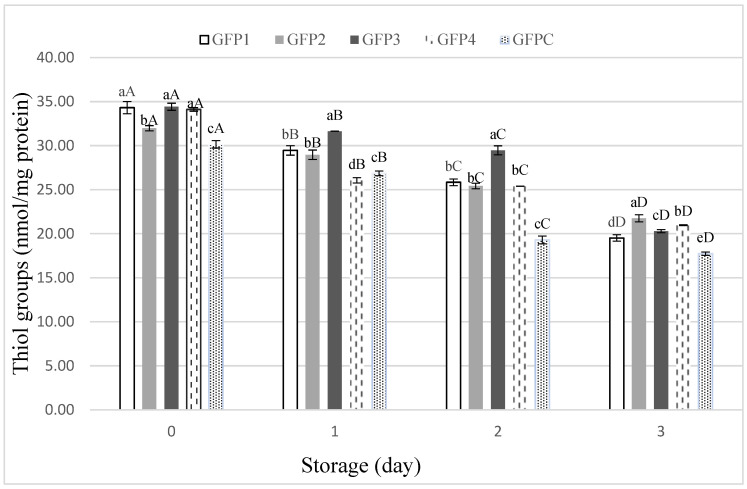
Protein thiol content of ground fish patties during storage (Different lower cases in superscripts (a–e) indicate difference (*p* ≤ 0.05) between treatments; Different Upper cases superscripts (A–D) indicate difference (*p* ≤ 0.05) between days of storage).

Oxidation of proteins is a critical factor affecting fish product quality, leading to structural alterations, impaired functionality, and deterioration of sensory characteristics. [42]. Oxidative modifications of proteins may result directly from reactive oxygen and nitrogen species, or indirectly through reactions with lipid oxidation products, reducing sugars, or carbohydrates [40]. Oxidative changes in cysteine residues result in protein carbonyl formation and disulfide bond cross-linking, which ultimately affect protein structure and key quality parameters, including tenderness and texture [43]. The extent of protein oxidation in ground fish patties was assessed by measuring thiol group content over three days of storage (Figure 3). As storage progressed, a significant decline (*p* ≤ 0.05) in thiol groups was observed across all samples, indicating the protein oxidation. Among the treated samples, GFP2 and GFP4 retained higher thiol contents compared to other treated samples. The protective effect of basil EO and LE against process of protein oxidation is linked to the presence of bioactive compounds, especially phenolic monoterpenes [44]. The hydroxyl group, in their structure, connected to the aromatic ring is capable of donating an electron to neutralize free radical reactions [28]. This correlates with their effectiveness in inhibiting lipid oxidation, as observed in the TBARS results. The observed correlation between protein and lipid oxidation emphasizes the interconnectedness of these oxidative processes in fish products. By-products of lipid oxidation, including reactive aldehydes, can directly react with proteins, accelerating thiol loss and promoting carbonyl formation [42,45]. The fact that treatments that effectively suppressed lipid oxidation also exhibited lower protein oxidation supports this mechanistic link. Overall, the results demonstrate that basil EO and LE effectively slow oxidative deterioration in ground fish patties, with GFP2 emerging as the most promising formulation. Although direct comparison with synthetic antioxidants such as BHT was not performed in this study, the observed reduction in lipid and protein oxidation highlights the potential of basil-derived extracts as effective natural antioxidants within clean-label fish formulations.

### 3.5. TVB-N Profile of Clean Label Ground Fish Patties

**Figure 4:**
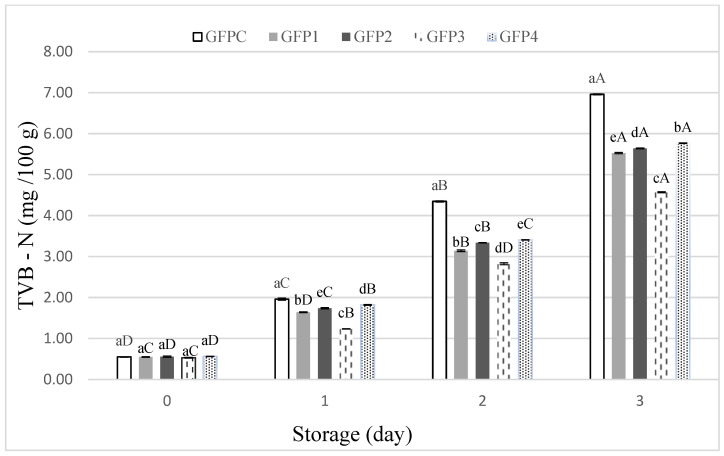
Total volatile base nitrogen content of ground fish patties during storage (Different lower cases in superscripts (a–e) indicate difference (*p* ≤ 0.05) between treatments; Different Upper cases superscripts (A–D) indicate difference (*p* ≤ 0.05) between days of storage).

Total volatile basic nitrogen (TVB-N) is widely used as an indicator of fish muscle spoilage, reflecting the accumulation of nitrogenous compounds generated by microbial and enzymatic activity. Elevated proteolytic activity increases the concentration of muscle-derived nitrogen, small peptides, and amino acids, while reducing protein solubility and altering functional properties. As shown in Figure 4, TVB-N levels increased across all treatments during storage. Initially, TVB-N values were low in all samples, indicating the freshness of the ground fish patties. However, as storage progressed, a significant increase (*p* ≤ 0.05) was observed, with the GFPC consistently showing the highest values. This suggests that the treatments (GFP1, GFP2, GFP3, and GFP4) effectively delayed protein breakdown and microbial activity. The effectiveness of basil EO and LE can be attributed to their bioactive compounds. Basil EO contains high levels of oxygenated monoterpenes such as linalool (66.95%) and eucalyptol (6.50%), which have strong antimicrobial properties, while LE is rich in sesquiterpene hydrocarbons like γ-cadinene (2.55%) and germacrene D (3.55%), which also contribute to microbial inhibition [46,47,48]. These results indicate delayed spoilage, as reflected by reduced accumulation of TVB-N. Among the treated groups, GFP3 outperformed other treatments, suggesting optimal efficacy in maintaining quality.

These results are consistent with the TBARS and protein oxidation findings, further supporting the role of these treatments in extending the shelf life of clean label ground fish patties. While longer storage periods are commonly applied in extended shelf-life studies, the selected 3-day storage period is particularly relevant for fresh fish patties and pilot-scale product development, where quality deterioration occurs rapidly. Future studies may extend storage duration or include modified atmosphere packaging to further explore long-term preservation effects.

## 4. Conclusions

Extracts of basil (*Ocimum basilicum* L.) prepared via hydrodistillation (HD) and supercritical fluid extraction (SFE) were evaluated as natural antioxidants in ground fish patties. SFE, a sustainable technique, yielded terpenoid-rich lipid extracts with notable antioxidative properties. Incorporating both EO and LE significantly mitigated lipid and protein oxidation and improved color stability while maintaining stable pH and water activity.

The most pronounced antioxidative effect was observed in patties treated with 0.150 µL/g of LE obtained by SFE. These findings support the use of basil-based extracts as potential “green” additives for extending shelf life and preserving the quality of fish-based products. Future research should explore their potential as promising natural antioxidant options for clean-label seafood products, including their sensory impact and regulatory acceptance. Ground fish or surimi treated with basil (*Ocimum basilicum* L.) extracts as a biopreservative may also serve as a promising base for future fish-based innovative food products.

## Figures and Tables

**Table 1:** Basil EO and LE chemical profile analyzed by GC-MS.

	Sample		EO		LE		
	Compound	RT (min)	Relative Percentage (%)	Std	Relative Percentage (%)	Std	Identification Method
1	α-Thujene	3.735	0.04	0.00	/	/	NIST, AI (924)
2	α-Pinene	3.868	0.27	0.02	0.08	0.00	NIST, AI (932)
3	Camphene	4.159	0.04	0.00	/	/	NIST, AI (946)
4	Sabinene	4.678	0.08	0.00	0.05	0.00	AI (969)
5	β-Pinene	4.757	0.51	0.00	0.19	0.01	NIST, AI (974)
6	Myrcene	5.07	0.20	0.00	0.07	0.01	NIST, AI (988)
7	α-Terpinene	5.721	0.07	0.01	/	/	NIST, AI (1014)
8	*p*-Cymene	5.959	0.07	0.01	0.03	0.00	NIST, AI (1020)
9	Limonene+β-Phellandrene	6.065	0.10	0.03	0.05	0.01	NIST, AI (1024/1025)
10	Eucalyptol (1,8-Cineole)	6.129	6.50	0.03	3.68	0.04	NIST, AI (1026), ST
11	Lavender lactone	6.409	/	/	0.28	0.03	AI (1034)
12	*trans*-β-Ocimene	6.642	0.07	0.01	/	/	NIST, AI (1044)
13	γ-Terpinene	6.965	0.15	0.01	0.05	0.00	NIST, AI (1054)
14	*cis*-Sabinene hydrate	7.23	0.06	0.01	0.21	0.01	NIST, AI (1065)
15	*cis*-Linalool oxide (furanoid)	7.378	0.87	0.03	2.25	0.02	NIST, AI (1067)
16	*trans*-Linalool oxide (furanoid)	7.855	0.70	0.04	2.00	0.03	NIST, AI (1084)
17	Linalool	8.268	66.95	0.66	60.80	0.27	NIST, AI (1095)
18	Camphor	9.777	0.74	0.02	0.61	0.01	NIST, AI (1141)
19	Borneol	10.656	0.24	0.02	0.17	0.01	NIST, AI (1165)
20	Epoxylinalol	10.804	/	/	0.28	0.01	NIST
21	Menthol	10.947	0.10	0.01	/	/	NIST, AI (1167)
22	Terpinen-4-ol	11.043	0.66	0.03	0.55	0.00	NIST, AI (1174)
23	α-Terpineol	11.599	0.71	0.01	0.35	0.05	NIST, AI (1186)
24	2,6-Dimethyl-3,7-octadiene-2,6-diol	11.699	/	/	1.06	0.02	NIST
25	Estragole	11.863	0.75	0.03	0.69	0.01	NIST, AI (1195)
26	Carvone	13.664	/	/	0.09	0.01	NIST, AI (1239)
27	Geraniol	14.214	0.44	0.03	0.65	0.02	NIST, AI (1249), ST
28	Bornyl acetate	15.29	0.29	0.01	0.24	0.01	NIST, AI (1284)
29	1,5,5-Trimethyl-6-methylene-cyclohexene	17.302	0.07	0.01	0.12	0.00	NIST
30	α-Cubebene	17.815	0.09	0.00	0.10	0.00	NIST, AI (1345)
31	Eugenol	18.377	0.73	0.05	2.82	0.07	NIST, AI (1356)
32	α-Copaene	18.832	0.18	0.02	0.28	0.01	NIST, AI (1374)
33	β-Elemene	19.52	1.94	0.03	2.58	0.02	NIST, AI (1389)
34	*trans*-Caryophyllene	20.495	0.32	0.01	0.59	0.02	NIST, AI (1417)
35	β-Copaene	20.956	0.08	0.01	0.12	0.01	NIST, AI (1430)
36	α-*trans*-Bergamotene	21.252	1.83	0.05	1.98	0.06	NIST, AI (1432)
37	α-Guaiene	21.406	0.55	0.04	0.93	0.02	NIST, AI (1437)
38	α-Humulene (α-Caryophyllene)	21.856	0.37	0.01	0.46	0.01	NIST, AI (1452)
39	*cis*-Muurola-4(14),5-diene	22.263	0.27	0.01	0.38	0.00	NIST
40	Germacrene D	22.962	1.71	0.06	3.55	0.01	NIST, AI (1484), ST
41	Bicyclogermacrene	23.54	0.37	0.00	0.62	0.01	NIST, AI (1500)
42	α-Bulnesene (δ-Guaiene)	23.979	0.90	0.00	1.59	0.02	NIST, AI (1509)
43	γ-Cadinene	24.292	1.73	0.09	2.55	0.05	NIST, AI (1513)
44	*trans*-Calamenene	24.642	0.13	0.01	0.14	0.03	NIST, AI (1521)
45	δ-Cadinene	24.715	0.27	0.03	0.20	0.02	NIST, AI (1522)
46	Spathulenol	26.706	0.81	0.06	0.58	0.01	NIST, AI (1577)
47	Cubenol <1,10-di-epi->	28.099	0.80	0.01	0.62	0.01	NIST, AI (1617)
48	τ-Cadinol	29.084	5.19	0.23	4.06	0.05	NIST, AI (1638)
49	α-Cadinol	29.597	0.25	0.01	0.29	0.02	NIST, AI (1652)
	Monoterpene hydrocarbons		1.59		0.52		
	Sesquiterpene hydrocarbons		10.71		16.06		
	Oxygenated monoterpenes		79.72		75.38		
	Oxygenated sesquiterpenes		7.04		5.55		
	Other		0.07		1.46		
	Total		99.23		99.05		

RT—retention time, EO—essential oil, LE—lipid extract, Std—standard deviation.

**Table 2:** Quantitative content of dominant terpenoid compounds from basil EO and LE.

	EO		LE	
Compound	mg/g	Std	mg/g	Std
Eucalyptol (1,8-Cineole)	36.65	1.09	12.21	0.05
Germacrene D	15.99	0.14	6.3	0.01
Geraniol	10.38	0.04	14.97	0.05

EO—essential oil, LE—lipid extract, Std—standard deviation.

**Table 3:** Mean pH and aw values of control and treated ground fish patties during storage period at 4 °C.

	**pH Values**				
**Storage day**	Treatments				
	GFPC	GFP1	GFP2	GFP3	GFP4
**0**	5.97 ± 0.06 ^aA^	5.99 ± 0.05 ^abA^	6.13 ± 0.02 ^bB^	5.98 ± 0.02 ^aA^	6.23 ± 0.01 ^bC^
**1**	6.03 ± 0.01 ^abA^	6.06 ± 0.02 ^bcA^	6.03 ± 0.03 ^aA^	6.05 ± 0.01 ^aA^	6.04 ± 0.01 ^aA^
**2**	6.08 ± 0.04 ^bA^	6.10 ± 0.01 ^cA^	6.06 ± 0.02 ^abA^	6.08 ± 0.05 ^aA^	6.06 ± 0.01 ^aA^
**3**	6.01 ± 0.03 ^abA^	5.96 ± 0.05 ^aA^	6.08 ± 0.08 ^abA^	6.05 ± 0.11 ^aA^	6.00 ± 0.07 ^aA^
	**a_w_ Values**				
**Storage day**	Treatments				
	GFPC	GFP1	GFP2	GFP3	GFP4
**0**	0.959 ± 0.001 ^aA^	0.964 ± 0.001 ^aB^	0.964 ± 0.001 ^aB^	0.968 ± 0.001 ^cC^	0.958 ± 0.001 ^aA^
**1**	0.961 ± 0.004 ^aA^	0.962 ± 0.001 ^aA^	0.965 ± 0.006 ^abA^	0.965 ± 0.001 ^aA^	0.961 ± 0.004 ^bA^
**2**	0.966 ± 0.001 ^bB^	0.960 ± 0.006 ^aA^	0.964 ± 0.001 ^aAB^	0.961 ± 0.002 ^bA^	0.963 ± 0.001 ^cAB^
**3**	0.965 ± 0.001 ^bA^	0.969 ± 0.002 ^bB^	0.969 ± 0.002 ^bB^	0.964 ± 0.001 ^aA^	0.966 ± 0.001 ^dA^
	L* Values				
**Storage day**	Treatments				
	GFPC	GFP1	GFP2	GFP3	GFP4
**0**	71.43 ± 3.55 ^aA^	69.47 ± 2.04 ^aA^	71.32 ± 1.35 ^abA^	72.57 ± 2.97 ^aA^	70.55 ± 2.40 ^aA^
**1**	68.62 ± 3.89 ^aA^	69.40 ± 7.66 ^aA^	70.66 ± 2.97 ^aA^	70.27 ± 1.39 ^aA^	71.80 ± 3.34 ^aA^
**2**	71.43 ± 3.57 ^aA^	71.43 ± 3.56 ^aAB^	71.43 ± 3.57 ^aAB^	71.43 ± 3.58 ^bAB^	72.04 ± 3.01 ^aB^
**3**	71.78 ± 0.88 ^aA^	70.73 ± 1.22 ^aA^	73.54 ± 1.19 ^bB^	71.98 ± 0.65 ^aA^	71.70 ± 1.54 ^aA^
	a* Values				
**Storage day**	Treatments				
	GFPC	GFP1	GFP2	GFP3	GFP4
**0**	2.10 ± 2.62 ^aA^	2.31 ± 1.31 ^aA^	1.33 ± 0.42 ^aA^	1.52 ± 0.65 ^aA^	1.89 ± 0.94 ^aA^
**1**	0.75 ± 0.57 ^aA^	1.92 ± 1.45 ^aA^	0.72 ± 0.85 ^aA^	0.64 ± 0.71 ^aA^	1.58 ± 1.29 ^aA^
**2**	0.12 ± 0.58 ^abAB^	0.06 ± 0.44 ^aA^	0.76 ± 0.49 ^aBC^	0.52 ± 0.47 ^aABC^	1.13 ± 0.33 ^aC^
**3**	0.75 ± 0.35 ^aAB^	0.17 ± 0.25 ^aA^	1.08 ± 0.38 ^aB^	0.56 ± 0.58 ^aAB^	1.83 ± 0.46 ^aC^
	b* Values				
**Storage day**	Treatments				
	GFPC	GFP1	GFP2	GFP3	GFP4
**0**	8.46 ± 2.09 ^abA^	8.28 ± 1.03 ^aA^	8.06 ± 0.74 ^aA^	8.60 ± 1.00 ^aA^	9.12 ± 0.87 ^aA^
**1**	9.37 ± 2.08 ^abA^	15.61 ± 6.67 ^bB^	8.32 ± 2.29 ^aA^	9.78 ± 1.95 ^aA^	10.35 ± 1.28 ^aA^
**2**	6.95 ± 2.95 ^aB^	8.55 ± 1.31 ^aAB^	10.23 ± 1.32 ^bA^	8.49 ± 1.95 ^aAB^	10.47 ± 1.24 ^aA^
**3**	11.31 ± 0.72 ^bAB^	10.11 ± 1.14 ^aA^	12.61 ± 0.54 ^cB^	10.37 ± 2.68 ^aA^	12.47 ± 1.06 ^bB^
	**Δ*Ε* Values**				
**Storage day**	Treatments				
	GFPC	GFP1	GFP2	GFP3	GFP4
**0**	-	8.79 ± 0.95 ^bC^	1.57 ± 0.67 ^bAB^	2.33 ± 2.34 ^aA^	2.58 ± 0.81 ^aA^
**1**	-	9.26 ± 8.18 ^bB^	3.48 ± 1.68 ^aA^	3.06 ± 1.02 ^bA^	3.80 ± 1.18 ^abAB^
**2**	-	2.72 ± 0.50 ^aA^	3.34 ± 0.46 ^aA^	4.75 ± 0.33 ^abAB^	3.33 ± 1.90 ^abA^
**3**	-	3.01 ± 0.38 ^aA^	4.88 ± 0.75 ^cC^	3.55 ± 0.39 ^abAB^	4.28 ± 1.00 ^bCB^

Means ± SD with different letters (A–C) in the same row are significantly different (*p* ≤ 0.05) Values with different letters (a–d) in the same column are significantly different (*p* ≤ 0.05). GFPC: Ground fish patties with no basil EO and LE. GFP1: Ground fish patties containing 0.075 µL/g basil EO. GFP2: Ground fish patties containing 0.150 µL/g basil EO. GFP3: Ground fish patties containing 0.075 µL/g basil LE. GFP4: Ground fish patties containing 0.150 µL/g basil LE.

## Data Availability

The original contributions presented in this study are included in the article. Further inquiries can be directed to the corresponding authors.
